# The Role of NLRP3 Inflammasome in the Pathogenesis of Stevens-Johnson Syndrome/Toxic Epidermal Necrolysis

**DOI:** 10.18103/mra.v12i1.4939

**Published:** 2024-01-30

**Authors:** Mallika Shekhar, Omer Iqbal, Adarsh Dharan, Hanin El-Khateeb, Kavya Jatavallabhula, Ping Bu, Charles Bouchard

**Affiliations:** 1. Loyola University Stritch School of Medicine, Maywood, IL. 60153 USA.; 2. Departments of Ophthalmology & Pathology, Loyola University Stritch School of Medicine, Maywood, IL.60153; 3. Department of Microbiology & Immunology, Loyola university Stritch School of Medicine, Maywood, IL. 60153; 4. Department of Ophthalmology, Loyola University Medical Center, Maywood, IL. 60153 USA.

## Abstract

Stevens Johnson Syndrome/Toxic Epidermal Necrolysis (SJS/TEN) are mainly drug-induced severe cutaneous adverse reactions with increased mortality. It also involves the eyes causing ocular surface disease leading to visual impairment and blindness. The role of NLRP3 Inflammasome in causing ocular surface disease and keratinocyte apoptosis is not fully explored. This study is focused on determining the role of NLRP3 Inflammasome in the pathogenesis of Stevens Johnson Syndrome/Toxic Epidermal Necrolysis. The NLRP3 Inflammasome plays a crucial role in the pathogenesis of Stevens Johnson Syndrome/Toxic Epidermal Necrolysis and may correlate with the degree of severity of skin detachment and ocular surface disease. This study looked at the expression of the NLRP3 Inflammasome in the skin of patients with biopsy confirm Stevens Johnson Syndrome/Toxic Epidermal Necrolysis compared to the lichen planus and normal controls by immunohistochemistry as well as observing the mitochondrial function of platelets challenged with plasma from patients with Stevens Johnson Syndrome/Toxic Epidermal Necrolysis and Normal Human Plasma using Agilent Seahorse XF Analyzer. Under a current, Loyola IRB approved protocol, 12 collected and archived unstained slides of skin and blood plasma samples from patients with biopsy confirmed Stevens Johnson Syndrome/Toxic Epidermal Necrolysis was used for this study. Immunohistochemical analysis was performed using anti-NLRP3 antibodies followed by imaging on a Delta Vision microscope. The precise roles of cytokines and chemokine receptors in severity of skin detachment has not been completely studied. The identification of the roles of NLRP3 in Stevens Johnson Syndrome/Toxic Epidermal Necrolysis would bridge the gaps in the basic understanding regarding the pathogenesis of this disease spectrum. NLRP3 Inflammasome is a potential therapeutic target and its inhibition by phytochemicals may be appropriate effective treatment strategies in the management of this condition.

## Introduction

Stevens-Johnson Syndrome and Toxic Epidermal Necrolysis (SJS/TEN) are generally classified as Severe Cutaneous Adverse Reactions (SCARs) and are rare life-threatening severe mucocutaneous lesions leading to necrosis and sloughing of the skin. They are often associated with multiorgan system involvement leading to hematological, ophthalmological and genitourinary complications. Stevens Johnson Syndrome/Toxic Epidermal Necrolysis exhibit symptoms of extensive blistering rash, high body temperature, and damage to the mucous membranes. Stevens Johnson Syndrome/Toxic Epidermal Necrolysis are considered to be similar conditions characterized by the detachment and blistering of the skin in various areas of the outer layer^24,30–33^. These reactions are exceptionally uncommon, occurring in approximately 1 to 5 cases per year per 1 million individuals, and typically occur within a few days to weeks after starting the medication. Individuals affected by SJS/TEN require hospitalization, and the mortality rates are relatively high, with SJS having a mortality rate of 1 to 5% and TEN having a mortality rate of 20 to 30%. Even after recovering from the condition, patients may experience long-term complications, such as vision loss caused by the formation of a pseudomembranes^16^.

The pathogenesis of this condition is not fully understood. There have been case studies done that reported a high association between SJS/TEN and certain medications. In many populations, the risk of association is low, but there are special cases such as the link between a certain HLA increasing the risk of SJS/TEN in the Han Chinese population using carbamazepines^14^. Several strong associations of prevalence of SJS/TEN in South-East Asia have been documented with associations of HLA-A*0206, HLA-B*44:03, HLA-B*1502 (carbamazepine) and HLA-B*5801 (allopurinol) genotypes^1–4^. Stevens Johnson Syndrome/Toxic Epidermal Necrolysis are believed to be immune-mediated specific hypersensitivity reactions initiated by cytotoxic T-lymphocytes^5,6^. Cytotoxic T cells and NK cells were found to infiltrate the skin lesions of subjects with SJS/TEN^7^. The involvement from CD8+ T cells and NK cells that show the potential of IL-15 to be involved in the disease pathology as well, as this cytokine works to promote T cell and NK cell development, survival, and stimulation to produce more inflammatory cytokines to further stimulate cytotoxicity^14^. Increased expression of the death cell receptor TNF-α or TNF receptor 1 has also been reported^8^. Recently our laboratories reported on the role of IL-13, IL-15 and granulysin in the pathogenesis of SJS/TEN^12^.

Innate immune cells and keratinocytes primarily house protein complexes called inflammasomes, which are integral to the innate immune response. Inflammasomes are a set of protein complexes located within the cytoplasm that act as detectors of innate immunity, triggered by the presence of invading pathogen-associated molecular patterns (PAMPs) or internal danger-associated molecular patterns (DAMPs)^18,34,44.^ Previous studies have indicated that mitochondrial damage, the innate immune response, inflammation, and cell death play crucial roles in the development and mechanisms of most drug-induced toxicity^18^. The NLRP3 inflammasome works as a sensor of potential infection and toxicity and is important in the pathogenesis of different conditions^18,25–29^. The NLRP3 inflammasome can be found in epithelial tissues and keratinocytes and is composed of three main components: nucleotide-binding domain-like receptor with pyrin-domain 3, apoptosis-associated speck-like protein (ASC), and pro-caspase-1.

The activation of the NLRP3 inflammasome can be triggered by various external stimuli^17,45^. Most drug induced toxicity resulting in skin diseases are seen to activate the NLRP3 inflammasome which causes a dysregulated inflammatory response. For example, Carbamazepine which is used to treat epilepsy is seen to activate the NLRP3 inflammasome which causes the dysfunction of the inflammatory response in patients resulting in SJS/TEN. Zhang et al. completed a study assessing how a metabolite of carbamazepine activated the NLRP3 inflammasome in keratinocytes in SJS/TEN. They collected blister fluid from patients with carbamazepine induced SJS/TEN and analyzed them using ELISA and PCR. They saw that carbamazepine resulted in an increased expression of NLRP3 inflammasome in the patients with SJS/TEN that led to other downstream activation and cytotoxic functioning of CD8+ T lymphocytes in the skin which additionally contributes to the pathology of SJS/TEN^15^. Some other examples are Imiquimod which causes psoriasis like symptoms, Nevirapine which causes skin rashes, and Telaprevir/Boceprevir which cause cutaneous adverse effects all through the activation of the NLRP3 inflammasome and other cytokines causing cell damage and increased inflammation^18^. This activation of NLRP3 inflammasome is due to the overproduction of ROS and NF-κB transcription^18,36^. While the exact process of NLRP3 activation remains uncertain, researchers have proposed three potential molecular mechanisms. These include the efflux of potassium ions (K+), signaling involving the production of reactive oxygen species (ROS), mitochondrial dysfunction, and a third model that relies on the lysosome’s rupture^19^.

Furthermore, many studies have been done that look at the association between mitochondria and the activation of the inflammasome. Mitochondria can produce ROS which is a key component of activating the NLRP3 inflammasome^20^. Studies done by Shimada et al. found a significant role of mitochondrial dysregulation, production of mtROS, and mtDNA in activation of the NLRP3 inflammasome causing other downstream signaling effects that ultimately lead to pyroptosis^20^.

NLRP3 Inflammasome is a promising therapeutic target for drug-induced toxicity^13^. Nucleotide-binding oligomerization domain (NOD)-like receptor family pyrin domain containing 3 (NLRP3) Inflammasome, serving as a sensor of infections and external stimuli, are involved in the pathological processes of various diseases^13^. NLRP3 Inflammasome is reported to be involved in the initiation and deterioration of drug-induced toxicity of multiple organs such as liver, kidney and the heart through multiple signaling pathways^13^. Its role in the involvement of skin and eyes has not been studied. Given that SJS/TEN is mostly drug-induced and partly due to infections, often involving the skin, eyes and multiple organs, the role of NLRP3 Inflammasome in its pathogenesis will be of utmost importance and interest. This is one of the first studies that investigates both the role of NLRP3 inflammasome in the pathogenesis of SJS/TEN as well as how the NLRP3 inflammasome affects the mitochondrial functioning and oxygen consumption rates in platelets of patients with SJS/TEN.

## Methods

### Experimental design for skin biopsy analysis

Under a current, Loyola Institutional Review Board (IRB) approved protocol, 12 collected and archived unstained slides of skin from biopsy confirmed SJS/TEN patients and Lichen Planus patients as controls were used for this study. The sections of the skin biopsies on the slides were deparaffinized by washing three times with xylene for 5 minutes each. The slides were then washed twice in 100% ethanol for 2 minutes each, washed once in 95% ethanol for 5 minutes, and finally washed once in 70% ethanol for 5 minutes. After being rinsed with distilled water for 1 minute, the skin samples were washed for 5 minutes in a phosphate buffer solution (PBS). All skin biopsies were blocked with normal goat serum for 1 hour. The biopsies were then treated with NLRP3 primary antibody and incubated for 30 minutes at 4°C. Following incubation, the biopsies were washed 3 times with PBS, then treated with secondary anti-rabbit IgG and diamino-2 pheylindole (DAPI) antibodies and then incubated for 30 minutes. After washing with PBS, the skin biopsies were mounted on fluorogel and images were taken with a DeltaVision Microscope equipped with a digital camera. Exposure times were kept consistent for all samples. The skin biopsy slides of non-SJS/TEN patients such as Lichen Planus were used as a control group. These slides were prepared and analyzed using the methodology described above. Deconvolution immunofluorescence (IF) was performed on all slides using a DeltaVision microscope equipped with a digital camera. Exposure times and settings were kept constant for all samples. After imaging, fluorescent intensity sum per punctum was determined using Imaris^®^ software. Following the subtraction of background auto-fluorescence, fluorescence from all samples, the degree of IF above baseline was quantified using the surface function in the 594-nm channel. Statistical analysis was performed using Graph Pad Prism software.

### Experimental Design for the Agilent Seahorse XF Cell Mito Stress Test

The Agilent Seahorse XF Cell Mito Stress Test measured the oxygen consumption rate (OCR) of cells on the Seahorse XFe and XF Extracellular Flux Analyzers. In this experiment, platelets were used and stimulated with plasma from patients with either SJS/TEN or normal plasma. The experiment started by preparing the assay medium with supplemented Seahorse XF DMEM or RPMI medium, starting with 1 mM pyruvate, 2 mM glutamine, and 10 mM glucose. Three tubes containing oligomycin, FCCP, and rotenone/antimycin A were utilized. The tubes were resuspended with the prepared assay medium to solubilize the compounds and form the compound stock solutions. These compound stock solutions were used to create compound working solutions for loading into the injection ports on sensor cartridges. Working solutions of 2 to 3 mL were prepared for each compound in the assay medium. To prepare the Agilent Seahorse XF cell culture microplate for the assay, the Seahorse XF cell culture microplates were removed from a 37 °C CO2 incubator and the cells were examined under a microscope to confirm confluence. Then, the assay medium was removed from the water bath, and the cell culture growth medium in the cell culture microplate was replaced with a warmed assay medium using a multichannel pipette. The cell culture microplate was placed into a 37 °C non-CO2 incubator for 45 minutes to 1 hour before the assay. Afterward, the assay was run using the program Wave, and the instructions provided there will be followed. To analyze the data, the Seahorse XF Mito Stress Test Report Generator automatically calculated the Seahorse XF Cell Mito Stress Test parameters from the Wave data that has been exported to Excel.

## Results

NLRP3 Inflammasome is a well-studied cytokine that plays important roles in a variety of inflammatory diseases. To investigate the expression of NLRP3 Inflammasome in patients with SJS/TEN, LP, and normal control, we performed the experimentation outlined in the previous section. The results of those experiments are discussed below.

### NLRP3 Intensities in SJS/TEN, Normal Control, and Lichen Planus Tissue Samples

**Figure 1:**
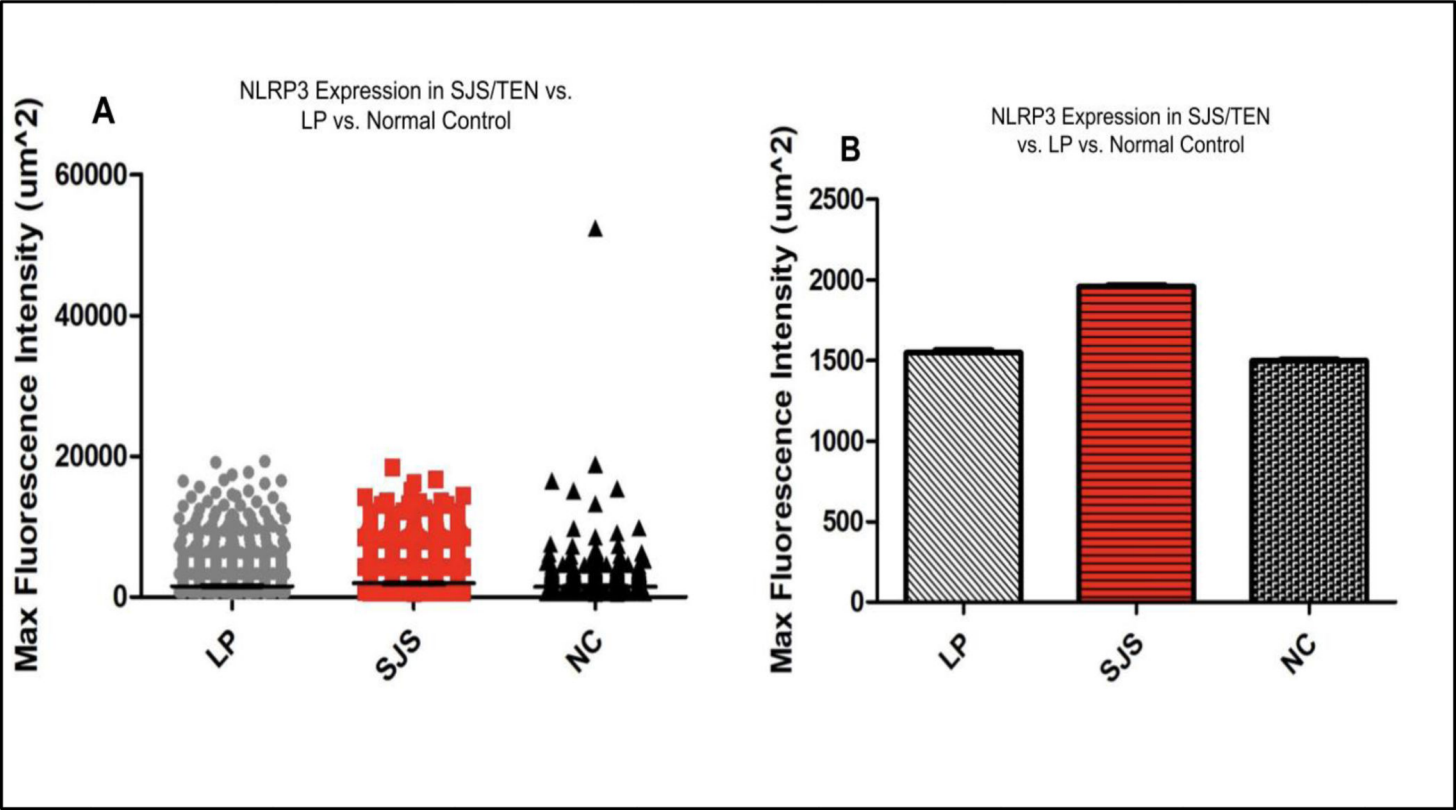
NLRP3 expression in LP, SJS/TEN, and Normal Control patient skin samples. Immunofluorescent puncta above baseline fluorescence for SJS/TEN patients were pooled and compared against pooled data from LP and Normal Control patients. The results are depicted above in both dot plot and bar graph formats. Experimentation revealed increased expression of NLRP3 in the skin of SJS/TEN patients compared to LP and Normal controls.

A total of 10 images each were taken from SJS/TEN, LP, and Normal Control skin biopsies and the samples were analyzed. All puncta for SJS/TEN patients above background auto-fluorescent intensity were pooled using a Kruskal-Wallis test. There was a significantly increased expression of NLRP3 inflammasome in the epithelium of pooled SJS/TEN patients (average IF intensity of 1958.163 um^2) compared to the epithelium of pooled LP patients (average IF intensity of 1547.783 um^2) and the epithelium of normal control patients (average IF intensity of 1496.824 um^2) (p<0.0001) (Figure 1A, 1B).

### Agilent Seahorse XF Cell Mito Stress Test

The mitochondrial stress test was performed using platelets of SJS/TEN patients vs normal control vs PMA, the positive control. The results show that the OCR values are significantly higher in the “Normal Plasma” category (average of 10.72 pmol/min) compared to the “SJS Plasma” category (average of 1.24 pmol/min) as well as between the PMA (average of 26.63 pmol/min) and control (average of 6.42 pmol/min) groups. This indicates a difference in the metabolic activity or oxygen consumption rate between these conditions, showing that the positive control is working in the experiment and that the oxygen consumption rate is significantly inhibited in SJS/TEN as compared to the normal plasma.

## Discussion

Stevens Johnson Syndrome/Toxic Epidermal Necrolysis is a very rare but severely fatal autoimmune disease that has systemic effects including necrosis and sloughing of the skin, rash, damage to the mucous membrane, and hematological, ophthalmological and genitourinary complications. Many studies have investigated the pathogenesis of this disease and the importance of identifying different cytokines and their significance in SJS/TEN. The results of this study suggest that NLRP3 plays a key role in the pathogenesis of SJS/TEN as it shows a statistically significant increase in the level of expression in the skin of SJS/TEN patients compared to LP or normal control patients. Furthermore, the results of the mitochondrial stress test show that the oxygen consumption rate was significantly inhibited in the platelets treated with SJS plasma in comparison with the normal plasma. To our knowledge, (aside from recent work by Zhang et al.) reports of NLRP3 acting as disease mediators in SJS/TEN specifically have not been previously reported^15^.

NLRP3 inflammasome is seen to be activated and involved in chronic inflammation of many autoimmune diseases^35,46^. One such disease where researchers have seen the activation of NLRP3 is in Systemic Lupus Erythematosis. The study done by Mähönen et al. investigated the expression of the NLRP3 inflammasome in the skin of patients with cutaneous lupus erythematosus (CLE). NLRP3 inflammasome when induced by various exogenous stimuli then activates the ASC which facilitates the self-cleavage of pro-caspase-1, converting it into its active form, caspase-1. Caspase-1, in turn, breaks down pro-interleukin-1β and prointerleukin-18 cytokines, transforming them into their highly inflammatory active states in response to any infection or stressors^17^. This is the proposed mechanism in how NLRP3 is involved in the pathogenesis and causation of many autoinflammatory diseases such as SJS/TEN. This study also showed that NLRP1, NLRP3, and ASC were all expressed in the skin of patients that had cutaneous lupus erythematosus as well as in the normal control patients at a lower level^17^. This is also seen in our experiments, as the expression of NLRP3 inflammasome can be seen in all three groups, SJS/TEN, LP, and normal control patients, but to a significantly higher level in the SJS/TEN group. This can be attributed to the fact that cytokines and inflammasomes are present in normal cells to combat certain forms of cell death such as pyroptosis and promote tissue repair^21^. There are many different pathogens and infectious agents that can attack normal cells and therefore the NLRP3 inflammasome needs to be present in these cells to generate an immune response. Although the NLRP3 inflammasome is seen in normal patients, it is seen to a higher extent in the LP patients and even higher in the SJS/TEN patients, showing that NLRP3 inflammasome plays a significant role in the pathogenesis of patients with SJS/TEN.

The roles of ROS and mitochondria in NLRP3 inflammasome activation still remain unclear. It was suggested that ROS is a common signal for NLRP3 inflammasome activation and that lysosomal NADPH oxidase is the source of ROS production^20^. Yet, there have also been several studies conducted showing that NLRP3 inflammasome activation is not affected by genetic or pharmacological inhibition of NADPH oxidase in human or animal cells^20^. Mitochondrial ROS also plays a role in the activation of NLRP3 inflammasome as studies have shown that mtROS, when produced by the inhibition of the mitochondrial respiratory chain, can activate the NLRP3 inflammasome^37–43^. Nakahira et al. found that the mtROS produced by dysfunctional mitochondria is needed for NLRP3 inflammasome activation^20^. Further, a study performed by Mu et al. focused on fluoxetine and other psychotropic drugs that work as exogenous DAMPs to directly trigger the NLRP3 inflammasome and its downstream signaling effects such as caspase activation. They also found that mitochondrial damage and mtROS accumulation were critical upstream molecular signals that resulted in the activation of fluoxetine induced NLRP3 inflammasome activation^23^.

Mitochondrial damage and mtROS accumulation are crucial molecular signals in drug-induced NLRP3 inflammasome activation. In our experiments, the results show that the oxygen consumption rate is significantly reduced in patients with SJS/TEN as compared to the normal control which is expected as the mitochondria is damaged in SJS/TEN, leading to more mtROS accumulation which causes a higher activation of the NLRP3 inflammasome in these patients along with a reduced oxygen consumption rate in their cells. SJS/TEN is a severe inflammatory skin condition that can affect multiple organ systems. This condition often leads to metabolic dysfunction, which can impact various physiological processes, including oxygen consumption. The reduced OCR in SJS plasma may be indicative of compromised metabolic function.

However, other studies have reported that ROS inhibitors also block the priming signal of the NLRP3 inflammasome activation and that mitochondrial dysfunction and mtROS production are inessential in NLRP3 inflammasome activation^20^. NLRP3 stimuli lead to various signaling and cellular processes and have been observed to initiate the activation of the NLRP3 inflammasome. Among these processes, K+ efflux is seen as the crucial signaling event necessary for NLRP3 inflammasome activation in response to most stimuli. However, the specific contributions of other events, like Ca2+ mobilization, Cl− efflux, ROS, and mitochondrial dysfunction, require further clarification^20^. Although our experiments support the role of mitochondrial dysfunction and mtROS production as a crucial activator of NLRP3 inflammasome, more research is needed to establish the role of ROS and mitochondrial dysregulation in NLRP3 activation.

Some limitations to our study include the small sample size as these skin biopsies were harvested from patients with SJS/TEN which is a very rare disease and therefore not that many biopsies can be obtained for analysis. Performing these experiments with a larger sample size would be beneficial to validate the results. Furthermore, platelets were used for the mitochondrial assay instead of human corneal endothelial cells. Blood flows invariably in all tissues including ocular tissues such the conjunctiva. The cornea does not receive its own blood supply and gets nutrients and various cytokines that influence the cornea through the aqueous humor. Since SJS/TEN involves the conjunctiva, and as the blood reaches these structures, platelets were utilized for the experiments as when they are activated, they can secrete various biomarkers like PDGF (platelet derived growth factor) and release various other cytokines including NLRP3 Inflammasome that may have a role in the pathogenesis of SJS/TEN. In future experiments, our lab will aim to culture the human corneal endothelial cells.

There are many areas of future research that still need to be explored when considering the implications of NLRP3 inflammasome in the pathogenesis of SJS/TEN. Based on these results, further studies could be done to stimulate these cells with individual cytokines involved in SJS/TEN in the presence or absence of inhibitors such as immunomodulatory agents to evaluate their therapeutic effects for patients. The study done by Mähönen et al. also showed a significant difference in the expression pattern produced by the NLRP3 inflammasome and its different components in patients with CLE, showing the pathogenesis of CLE as a heterogeneous disease with different subgroups of inflammation^17^. This can be an area of further research to see if SJS/TEN is also a heterogeneous disease as individual treatments would be needed depending on the specific subgroup which could be more beneficial in treating patients.

Additionally, research has been done on certain inhibitors of NLRP3 that can result in potential therapeutic effects for patients. Chen et al. performed a study to assess the role of NLRP3 on the pathogenesis of Huntington’s Disease, as well as the impact of an NLRP3 inhibitor. They used MCC950 which is a highly potent and selective inhibitor of NLRP3 that has been shown to inhibit the function of NLRP3 in other diseases such as Alzheimer’s in a transgenic mouse model of HD. Their results showed that a systematic administration of MCC950 was able to suppress the NLRP3 inflammasome in mice and decreased IL-1β, reactive oxygen species production, and neuronal toxicity. Furthermore, oral intake of MCC950 showed an increase in neuronal survival rate, reduction in inflammation, and improved motor function^22^. This experiment can provide a potential therapeutic treatment for other diseases as well such as SJS/TEN. Conducting research on the benefits of utilizing MCC950 to suppress the NLRP3 inflammasome in patients with SJS/TEN to see if there are any beneficial outcomes would be novel for future treatments for this fatal disease. Lastly, potential drug therapies could also target different mitochondrial enzymes and mtROS inhibition to further inhibit the downstream effects of NLRP3 inflammasome activation to see if they provide positive benefits for SJS/TEN patients. NLRP3 Inflammasome is a potential therapeutic target and its inhibition by phytochemicals may be appropriate effective treatment strategies in the management of this condition. Further studies could be done to stimulate keratinocytes and corneal epithelial cells with individual cytokines involved in SJS/TEN in the presence or absence of inhibitors such as immunomodulatory agents to evaluate their therapeutic effects for patients.

## Conclusion

Our results show a statistically significant increase in NLRP3 expression in the skin of SJS/TEN patients compared to LP and normal controls. Therefore, we conclude that NLRP3 inflammasome is a key regulator in the pathogenesis of SJS/TEN in the skin.

## Figures and Tables

**Figure 2:**
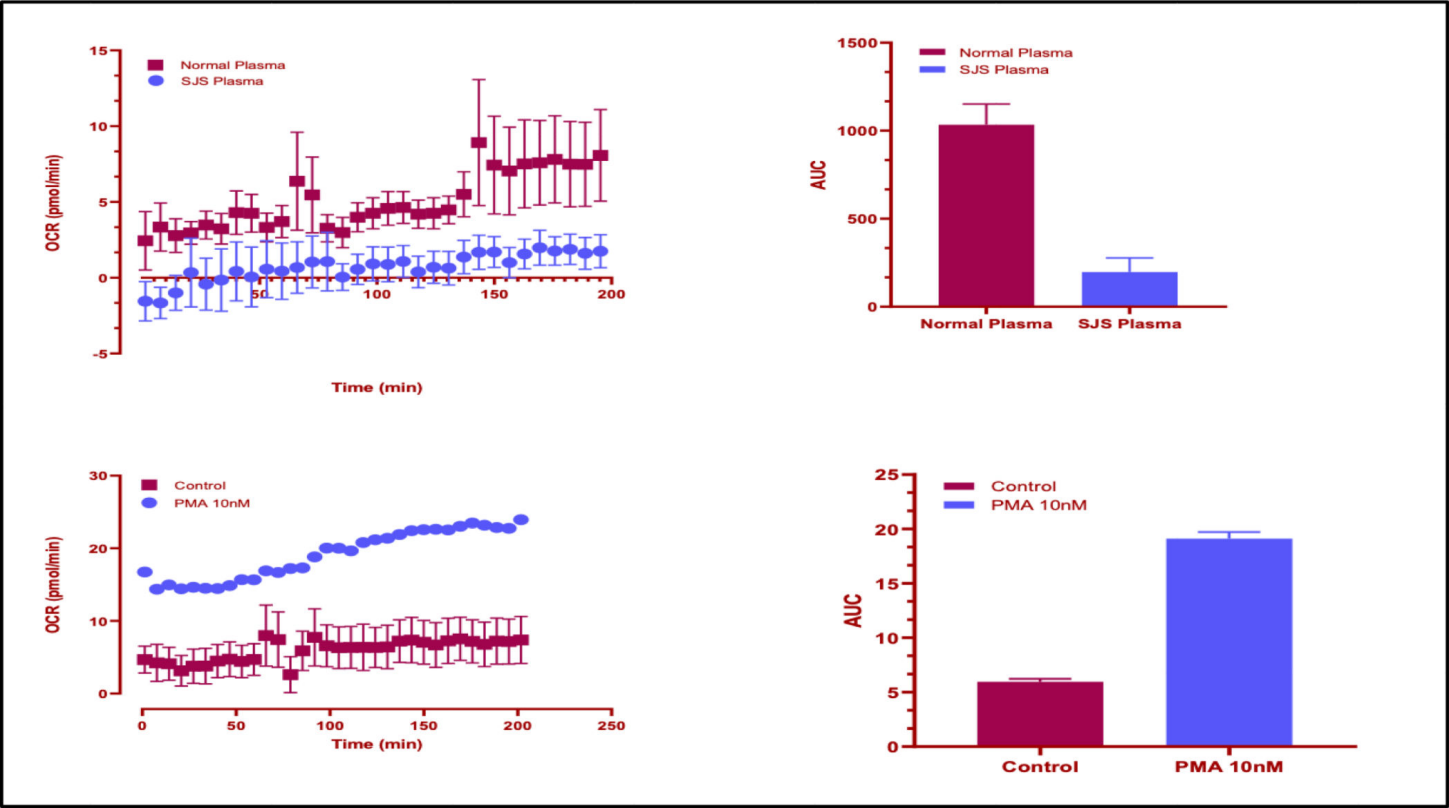
Agilent Seahorse XF Cell Mito Stress Test results of platelets of SJS/TEN patients vs normal control vs PMA, the positive control. The results are depicted above in both dot plot and an area under the curve bar graph formats. Experimentation revealed reduced oxygen consumption rates in the skin of SJS/TEN patients compared to normal controls.

**Figure 3:**
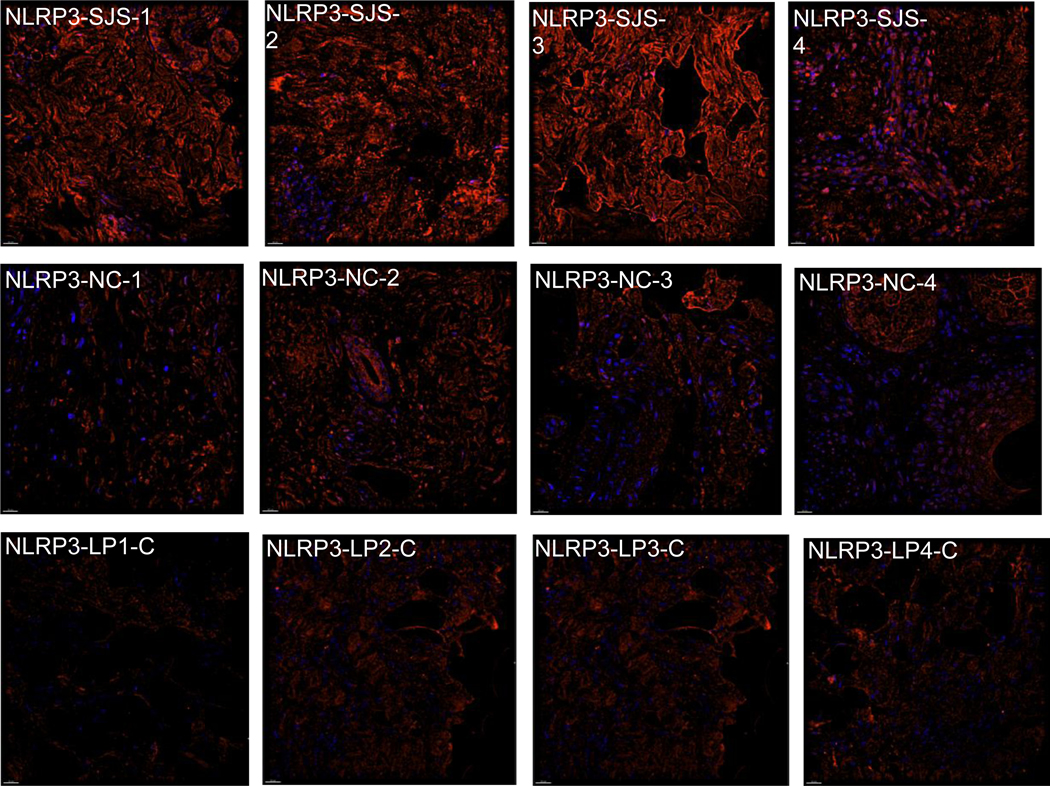
Representative images from Immunofluorescent (IF) microscopy taken with a DeltaVision Microscope equipped with a digital camera. Exposure times were kept consistent for all samples.
